# In Silico Evaluation of Algorithm-Based Clinical Decision Support Systems Based on Care Pathway Simulation Models: Scoping Review

**DOI:** 10.2196/72472

**Published:** 2026-03-24

**Authors:** Michael Dorosan, Ya-Lin Chen, Yan He, Qingyuan Zhuang, Sean Shao Wei Lam

**Affiliations:** 1 Health Services Research Centre, SingHealth, Academia, Ngee Ann Kongsi Discovery Tower Level 6, 20 College Road, Singapore, 169856, Singapore, 65 65767140; 2 Department of Biomedical Informatics and Medical Education, University of Washington, Seattle, WA, United States; 3 Department of Laboratory Medicine & Pathology, University of Washington Medicine, Seattle, WA, USA; 4 Operations Management, Lee Kong Chian School of Business, Singapore Management University, Singapore, Singapore; 5 OncoCare Cancer Centre, Singapore, Singapore; 6 Lee Kong Chian School of Business, Singapore Management University, Singapore, Singapore

**Keywords:** clinical decision support algorithms, *in silico* evaluation, ISE, clinical workflow simulation, health care modeling, digital twin, clinical decision, decision-making, decision support, workflow, support system, scoping review, algorithm-based, screening, thematic analysis, descriptive analysis, clinical decision-making

## Abstract

**Background:**

*In silico* evaluation (ISE) methods create a digital twin or a computer simulation of actual care pathways, enabling a broader assessment of the potential impact of algorithm-based clinical decision support systems (CDSS) before implementation. A programmatic search of several academic research databases showed at least 886 CDSS development and evaluation studies in the past 3 decades. However, fewer than 3% applied ISE to evaluate the potential impact on broader clinical care pathways.

**Objective:**

This study aims to review the scope of proposed ISE methods to evaluate CDSS, with a focus on simulation modeling approaches, care pathway parameters considered, and outcomes evaluated within the ISE methodological domain.

**Methods:**

This review followed the established scoping review methodological guidelines. We conceptualized a tailored search framework and conducted a 2-stage screening process on studies identified through automated searches of selected databases. Relevant information on CDSS study characteristics and the application of care pathway simulation modeling in CDSS evaluation was subsequently extracted.

**Results:**

A small subset of studies on CDSS development conducted ISE. Most ISE studies were published after 2019, reflecting a more recent increase in the application of ISE. These studies frequently emphasized patient, process, and cost-effectiveness outcomes. Notably, the evaluation of outcomes directly related to care providers’ well-being is lacking, highlighting a critical gap in current ISE applications. Among the studies included in this review, various simulation modeling paradigms were used, including dynamic simulations and state-based models. Three themes were found among the different motivations and objectives for using ISE: (1) outcome comparison, (2) outcome comparison with sensitivity analysis, and (3) simulation-based optimization of the proposed CDSS. The first two approaches considered a decoupled CDSS model training followed by simulation-based evaluation of the trained CDSS model. The third approach iteratively improved the CDSS model’s decision-support capabilities through optimization based on care pathway simulations. These approaches can be broadly categorized as *decoupled* (1 and 2), where CDSS models are evaluated within seperately designed clinical workflow simulations, and *integrated* (3), where simulation iteratively optimizes CDSS performance.

**Conclusions:**

The growing body of algorithm-based CDSS research underscores the need for evaluation approaches that are resource-efficient and account for systems-level workflow implications. This review highlights both the gaps and the potential of ISE, particularly care pathway simulation-based approaches, as a preimplementation strategy to strengthen evidence before significant resource allocations to pilot or full-scale implementations. ISE presents as a promising intermediary evaluation approach that bridges model-level performance and clinical workflow impact, allowing more contextualized and resource-efficient assessments prior to implementation.

## Introduction

Clinical decision support systems (CDSS) are widely used to improve point-of-care decision-making. These systems provide health care practitioners with timely information, reminders, and recommendations for patient care [1]. Such systems typically rely on standards of care, knowledge-based models, statistical methods, rule-based systems, machine learning (ML), and artificial intelligence (AI). The main objective is to support decision-making in diagnosis, prognosis, and care management processes [2,3]. Traditionally, they can be referred to as clinical scoring models [4] or clinical prediction rules [5,6]. Often, traditional CDSS are based on one or more risk factors that inform diagnosis, screening, and prognostication, or prescribe appropriate downstream care. While CDSS are grounded in evidence-based methods derived from limited observational datasets and clinical trials, the advent of AI and ML has recently enhanced CDSS’s ability to analyze large volumes of data quickly, offering greater insights and improving predictive capabilities [7-12].

Despite these advances, translating CDSS performance into real-world clinical impact remains challenging [13-17]. A persistent gap lies in the need to provide sufficient evidence of improvements across a broad set of outcomes [18,19]. Most evaluation frameworks emphasize discrimination metrics, such as precision, recall, area under the receiver operating characteristic, and area under the precision-recall curve. These metrics are often evaluated against historical ground-truth data [20-25]. However, high discrimination performance may not guarantee improved outcomes across stakeholders in the care pathway. The design of an appropriate threshold would also need to consider the implications for differing care pathways. The over-reliance on discrimination metrics assessed solely at the immediate CDSS decision point may fail to capture issues of generalizability, real-world use, clinical relevance, and safety in practice [22]. Recent studies have proposed moving beyond localized discrimination metrics toward more holistic outcome evaluations [19]. They have highlighted the extension of confusion matrix–based scores to net benefit measures that incorporate domain-specific tradeoffs [26,27] and emphasized usability, user trust, and workflow integration as essential indicators [23].

These observations underscore the need to consider system-level factors such as care pathways and resource constraints. For instance, a risk prediction model-based CDSS may fail to improve survival if intensive care unit beds, referral pathways, or staffing are limited. Similarly, thresholds for activating interventions may overwhelm available capacity or, conversely, miss patients who require timely care [26-28]. These challenges highlight the need for evaluation approaches that incorporate clinical workflows, operational constraints, and system-level effects.

**Figure 1.**
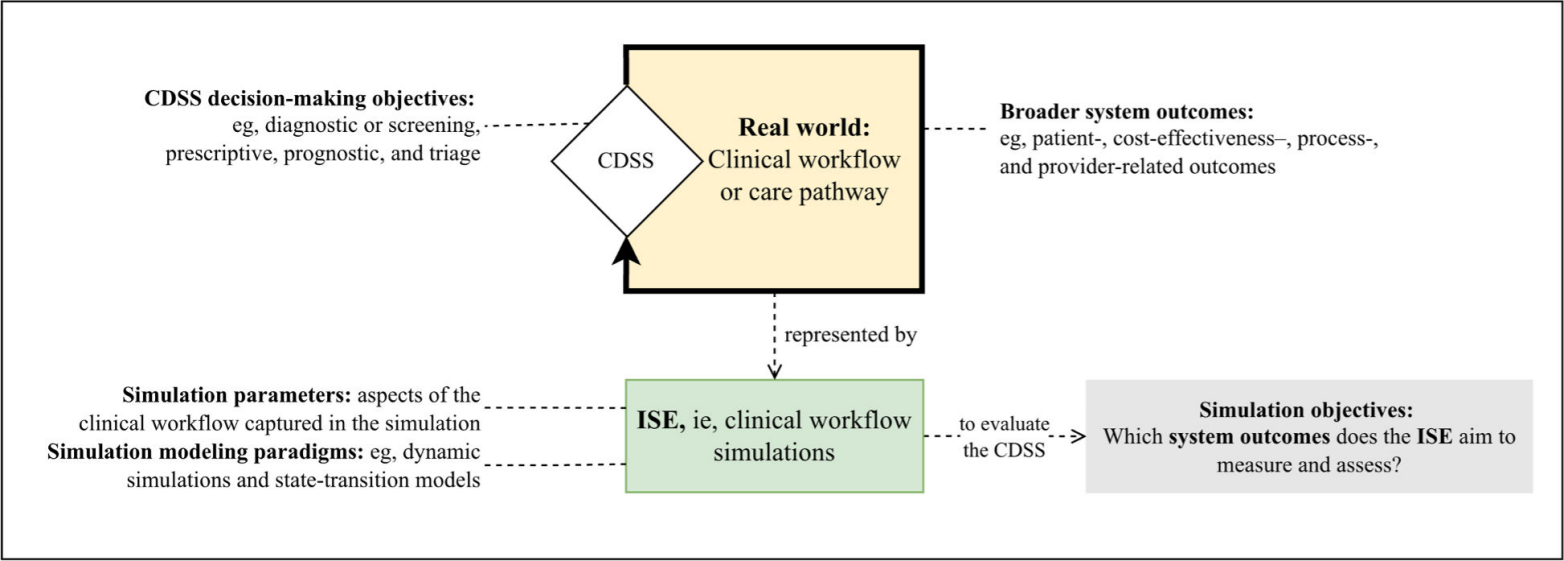
Illustration of key concepts in the scoping review and their relationships. This guided the development of the research questions for our review. CDSS: clinical decision support system; ISE: *in silico* evaluation.

Traditional approaches to assessing impact rely on clinical trials or pilot implementations [2]. These methods may be resource-intensive, difficult to scale, and inflexible to frequent updates and retraining, particularly in ML- or AI-based systems [3,29]. Similar tensions between evidence requirements and resource constraints exist in other biomedical domains, such as drug development, surgical systems innovation, and medical device evaluation. In these areas, *in silico* approaches that leverage computer-based representations of patient-level biological processes or cohort characteristics are already widely used to simulate actual clinical trials [30-35]. Our study draws on existing *in silico* methodologies by conceptualizing their integration with CDSS evaluation through simulated clinical workflows, as illustrated in Figure 1. This shifts the scope from simulated patients or cohorts to the capture of workflow dynamics, resource constraints, and operational dependencies when establishing evidence of potential impact [28,36,37].

In this review, we define *in silico* evaluation (ISE) as the use of simulated clinical care pathways and workflows with a computer program to evaluate a CDSS [28,30,36,38-41]. Existing methodological guidance on the CDSS development and evaluation lifecycle addresses potential impact assessment using decision-analytic measures and cost-effectiveness studies [3], clinical usefulness [5], silent evaluations [2,39], and effectiveness evaluation in a limited clinical setting [2]. We explore the role of ISE, which is not explicitly addressed within these existing development and evaluation frameworks.

Several studies have proposed ISE approaches for CDSS, ranging from domain-agnostic methods [42] to applications tailored to specific clinical decision-making tasks [43]. In parallel, numerous health care simulation guidelines from the Professional Society for Health Economics and Outcomes Research (ISPOR) provide methodological guidance for modeling and analysis. Despite these guidelines, the extent to which ISE has been applied in CDSS evaluation remains insufficiently characterized in reported studies.

With this background, we have formulated a scoping review to outline the scope and gaps of ISE for CDSS. We specifically aim to characterize the methodologies, outcome measures, and implementation contexts in which ISE is used to bridge the translational gap between CDSS model performance and real-world clinical impact (see the specific research questions [RQs] in Textbox 1). The conceptual framework guiding our scoping review is presented in Figure 1. How this framework was operationalized is detailed in the “Methods” section that follows.

Textbox 1. **Research questions (RQs) of this scoping review.**

The main RQ is as follows:

- What are the proposed *in silico* evaluation strategies for clinical decision support systems (CDSS) and the possible gaps in the reported studies?

Specific RQs for each included study in our scoping review:

- RQ1: What clinical domain and decision-making task does the CDSS aim to assist?
- RQ2: What *outcome* measures are used to evaluate potential impact?
- RQ3: What clinical workflow or pathway *parameters* are simulated by the study?
- RQ4: What simulation *modeling* paradigm is used?

## Methods

### Conceptual Framework

We followed the scoping review stages proposed by the Arksey and O’Malley framework [44], while considering recently proposed enhancements [45-47]. We also followed the scoping review guidelines outlined in the PRISMA-ScR (Preferred Reporting Items for Systematic Reviews and Meta-Analyses Extension for Scoping Reviews) checklist (Checklist 1) [46,48,49]. A common first step in scoping review guidelines is the formulation of a conceptual framework to guide the search, screening, and study selection.

We developed a tailored framework with 4 interrelated concepts that bound the scope of this review. Together, these four concepts identify studies that simultaneously: (1) propose a CDSS, (2) specify its decision-making objectives, (3) articulate an evaluative purpose, and (4) use an ISE-based strategy to generate evidence of potential impact. We operationalized the conceptual framework into searchable terms (Table 1 and Multimedia Appendix 1) and used them to guide automated search, screening, and data charting. Figure 1 illustrates how the four concepts intersect to define the target evidence base for this scoping review.

**Table 1.** Description of key concepts and keywords used to identify studies at the concepts’ intersection.

Key concepts	Description	Keywords
Clinical decision support models, algorithms, and systems	This concept concerns the underlying computational or statistical methods used to generate decision outputs for clinical care.	Machine learning, deep learning, artificial intelligence, reinforcement learning, supervised machine learning, unsupervised machine learning, semisupervised machine learning, self-supervised machine learning, expert system
Decision-making objectives of the CDSS^a^	This concept concerns the clinical purpose of the CDSS. Specifically, whether the system aids the following patient-level decision-making tasks: diagnosis, prognosis, screening, triage, prescribing, or other operational decisions.	Clinical decision support, clinical decision-making, prognosis, diagnosis, screening, triage
Objectives of the CDSS evaluation	This concept reflects why a CDSS is being evaluated. Relevant intents include validation, calibration, workflow impact analysis, decision analysis, and others. This concept ensures focus on those that explicitly assess performance, use, or potential impact.	Validation, potential impact, impact assessment, decision analysis, decision analytics measure, model calibration, model tuning, credibility, cost-benefit analysis
CDSS evaluation strategy	This concept identifies the approach used to evaluate the CDSS. The keywords indicate focus on ISE^b^ methods, those that use simulation modeling or digital twinning to represent clinical workflows to pre-implementation assessment of downstream effects.	In silico, computer simulation, digital twin, simulation, preimplementation, predeployment, computational simulation

a CDSS: clinical decision support system.

b ISE: *in silico* evaluation.

### Review Protocol

The steps in the review are described in the remaining subsections of the “Methods” section. For additional details on the methodology, the development of search strings, the pilot review process, and the specific operational definitions used in the review, readers are referred to our published study protocol [37].

We note the following deviations from our published research protocol [37]. First, during full data extraction, we operationalized the protocol’s questions for consistent coding by (1) integrating the “gaps” item into the main RQ, (2) clarifying RQ1-2 (domain or task; outcome measures), and (3) substituting the “objectives” item with concrete workflow parameters; the modeling-paradigm question was retained but reordered. Second, our inclusion and exclusion criteria were refined during the protocol implementation to improve clarity. Specifically, redundant items were consolidated, and inadequate descriptors, such as “purely methodological” and “system-level outcomes,” were reworded into broader, more operational terms for readability. These changes did not alter the scope of eligible evidence but contributed to a clearer basis for eligibility. Finally, the final data charting items were refined in accordance with the protocol to ensure consistent coding across heterogeneous studies. This is shown in the “Search Strategy and Screening” section.

### Search Strategy and Screening

Our review began with an automated search of selected databases in May 2023. Using multiple databases, including PubMed, Embase, CINAHL, PsycINFO, Cochrane, Web of Science, IEEEXplore, and arXiv, we programmatically searched across publications’ titles, abstracts, keywords, and Medical Subject Headings, as permitted by the databases’ automated search capabilities. The specific keywords and subject headings used are included in Multimedia Appendix 1.

Once the search results were collected, title and abstract screening were completed by November 2023. A subsequent full-text screening was conducted from December 2023 through May 2024. MD and YLC were the primary screeners of titles, abstracts, and full texts. Resolution of conflicts between them involved a series of group discussions with the entire study team, during which a unanimous vote was required to include studies that had been screened.

Studies were included if they addressed all 4 dimensions, thereby ensuring relevance to our research questions and positive operationalization of the core concepts. Specific examples include studies that: (1) enhance clinical decision-making for purposes such as diagnosis, triage, screening, prognosis, and prescribing; (2) use AI, automated algorithms, ML, or classical multivariate statistical methods; (3) evaluate CDSS models before deployment to gauge potential impacts; (4) use workflow simulation-based optimization in model development; (5) involve human participants; (6) include both experimental and observational studies, clinical and pragmatic trials, and pure validation studies; (7) are published in academic journals, conference proceedings, or preprint platforms; and (8) are written in English, with the publication year not being a limiting factor.

Our review excludes studies that: (1) do not involve clinical outcomes as one of their prediction targets; (2) evaluate only device-integrated models that automatically calculate and recommend actions without human-mediated interpretation; (3) apply ML or AI solely for descriptive analyses such as cluster analysis and cohort segmentation; (4) evaluate the accuracy of pathological specimens or sensor devices; (5) address system-level or population-level outcomes unrelated to direct patient-provider interactions; (6) rely solely on qualitative assessments; (7) are engineering in nature to improve medical data processing (eg, image enhancement, denoising, and image segmentation) without a defined application area or clinical prediction; (8) use conventional validation metrics (eg, area under the receiver operating characteristic, area under the precision-recall curve, and mean squared error), without addressing broader system-level use; (10) are related to proprietary systems that do not fully disclose the technology and algorithms; and (11) are literature review types (eg, scoping, systematic, rapid reviews, and meta-analyses). This list of excluded studies accounts for the conceptual boundaries of our review.

### Data Extraction and Operational Definitions

Data extraction began concurrently with the initial construction of the charting list and full-text screening, including a pilot trial. The data encoding database was completed in May 2024, followed by data analysis and the publication of the study protocol from July 2024 to February 2025. The final results and key findings are presented in this work.

A list of information to be extracted was developed based on our study objectives; this list was continuously refined as the team progressed through the review process. Reporting [38,39,50,51] and data extraction [52] guidelines for the conceptual framework shown in Table 1 informed the selection of relevant studies. Extracted variables are broadly categorized into: (1) study characteristics, (2) description of data used by the study, (3) decision-making objectives of the CDSS, and (4) details of the ISE conducted.

We define the ISE in our review as systems that simulate or represent clinical workflows using mathematical or computer-executed algorithms. Simulations aim to establish a “digital twin” of the actual clinical workflows or care pathways through a computer program [28,36,40,53]. They enable iterative evaluation of a CDSS across multiple scenarios in a resource-efficient, risk-free environment [28,36,41,42,54-56]. The design of these simulation models requires careful definition of three key components [55]: the outcomes to be measured, the simulation’s parameters, and the modeling paradigm.

*Outcomes* represent the effects of a CDSS within the workflow or care environment [18,21]. Examples of such outcomes include length of stay, adverse events, process throughput, delays, and cost reductions.

Simulation *parameters* capture the operational details of the simulated clinical workflow [56]. These include patient trajectories, arrival rates, provider adherence rates to CDSS recommendations, event durations, and resource constraints.

The *modeling paradigm* determines how the parameters interact to produce the observed outcomes. Well-known paradigms in simulation modeling include state-transition models [57] and dynamic simulation approaches [28] (eg, discrete-event simulation, agent-based modeling, and system dynamics). These are detailed in the “Results” section along with a mapping to the studies included in the review.

Table 2 presents specific items extracted from each included study. YLC, MD, SSWL, and QZ independently extracted information on study characteristics, data sources, and the CDSS’s decision-making objectives. Conflicts were resolved through discussions with the entire team. MD, YLC, and HY extracted details of the ISE conducted in each study, with SSWL and QZ acting as arbiters when necessary.

**Table 2.** Data charting and extraction items.

Data extraction broad concepts and items extracted		Details per item
Characteristics of studies included		
Authors		—^a^
Year of publication		—
Country of research		—
Title		Published study title
Data description		
Data source type		Examples: registry, health surveillance data, and institutional EHR^b^
Collection design		Examples: retrospective observational, prospective surveillance, and synthetically generated based on clinical trial data
Source name		Name of trials, registries, and hospitals where the data was sourced
Brief description		Narrative summarizing the data used by each study
Decision-making objectives of the CDSS^c^		
Domain of application		Examples: ED^d^, oncology, infectious diseases, and intensive care
Specific aim		Examples: disease risk, optimal treatment, mortality risk, cancer screening, and readmission risk
Aim classification (defined *a priori*)		Based on commonly cited CDSS task categories in literature [39,50,51,58]
ISE^e^ methods		
ISE objective		Examples: measure an outcome of interest with or without sensitivity analysis, and updating of CDSS
ISE evaluation outcome measures		Examples: length of stay, occurrence of a clinical event, cost reduction, referral rates, and provider burnout
ISE outcome theme classification (defined *a priori*)		High-level outcome groupings informed by existing review of CDSS studies as well as published development and reporting guidelines.
Reported simulation paradigm		Examples: discrete events simulation, agent-based modeling, Markovian state-transition models, and system dynamics simulations.
Simulation model parameters		Examples: Patient adherence, provider capacity, resource availability, interval between events, arrival rates, and procedure costs
Simulation model parameters theme classification (defined *a priori*)		Grouped according to high-level domains similar to outcome classifications
Simulation model scope description		A summary of the clinical workflow being simulated

a Not applicable.

b EHR: electronic health record.

c CDSS: clinical decision support system.

d ED: emergency department.

e ISE: *in silico* evaluation.

### Thematic Analysis

To synthesize heterogeneous findings, we conducted a thematic analysis of the CDSS decision-making objectives and the ISE method described in the included studies. Table 2 also cites related literature that informed the design of *a priori* themes used to classify studies by their specific clinical decision-making focus, ISE outcomes evaluated, and parameters included in the simulation. The thematic analysis approach for each data category is described below.

For CDSS decision-making objectives, we used a deductive approach based on commonly identified task classifications in prior CDSS reporting [39,50,51,58] and development guidelines [3,5], as well as reviews of CDSS studies [1]. These studies informed the *a priori* thematic grouping of decision-making objectives during data extraction.

For ISE outcomes, we drew on previously published CDSS reporting guidelines [39], methodological recommendations [3], and reviews [7,18,23,59] that distinguish among the types of impact measures commonly used in CDSS development studies. In addition, we considered workflow-related outcomes emphasized in health care simulation modeling literature [28,55] and in a review that evaluates CDSS success factors postimplementation. An *a priori* thematic grouping was similarly conducted for this topic.

For simulation modeling parameters, we used the same *a priori* analytic structure as ISE outcomes, as befitting the aspect of the care delivery system they represent. This ensured consistency in how we interpreted and compared simulation modeling inputs (ie, parameters) and outputs (ie, outcomes) across studies.

For simulation modeling paradigms, we recorded the specific simulation approach reported in each study and coded each according to standard definitions in health care simulation modeling literature [28,57,60,61]. Inductive coding was applied to define new thematic groupings. After all studies were reviewed, the team developed higher-level themes describing how simulation models were used in CDSS model development. All inductive categories were iteratively refined through team discussions and consensus.

## Results

### Overview

**Figure 2.**
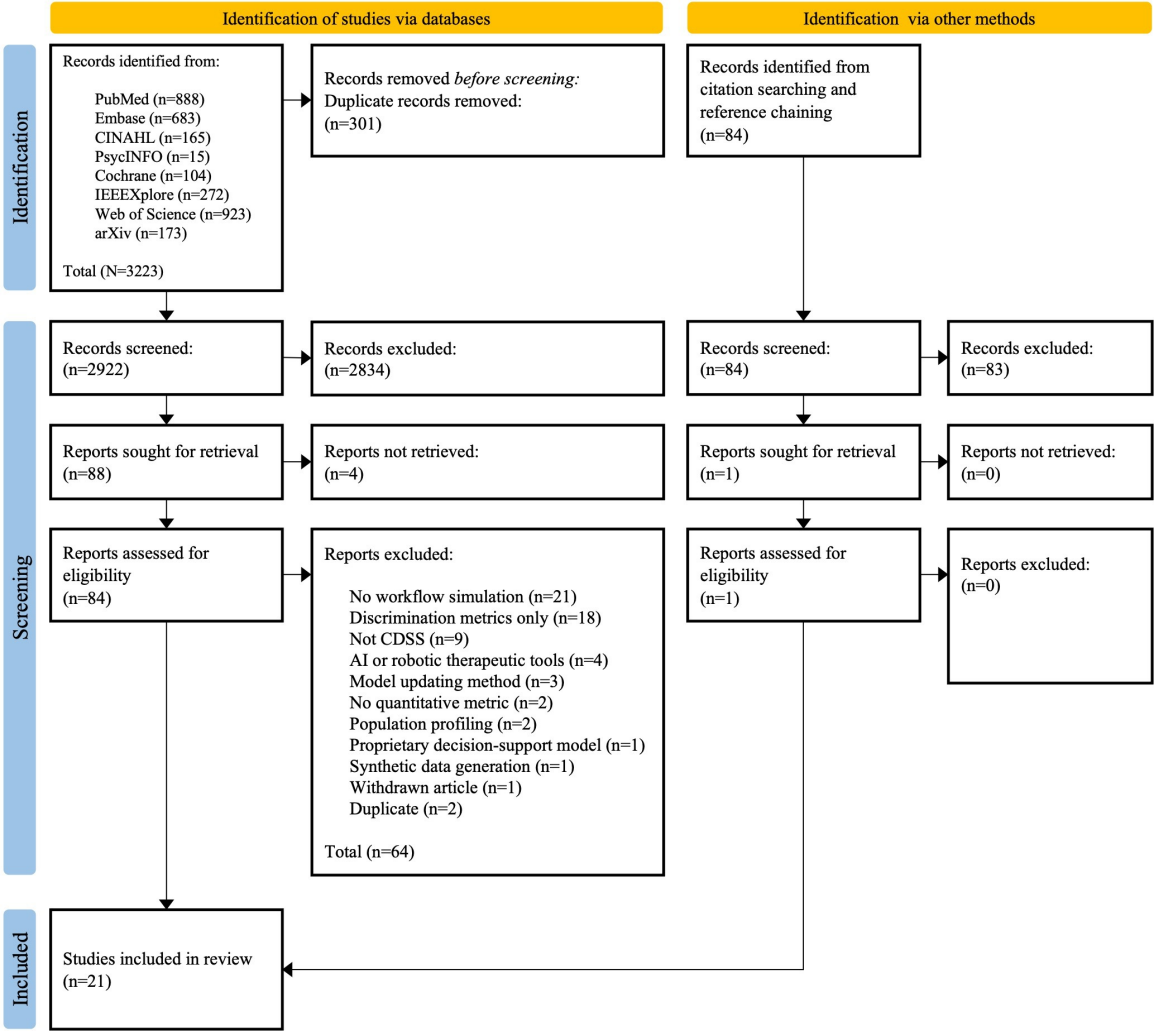
PRISMA-ScR (Preferred Reporting Items for Systematic Reviews and Meta-Analyses Extension for Scoping Reviews) flowchart. AI: artificial intelligence; CDSS: clinical decision support system.

A total of 3223 studies were collected from the included databases. The breakdown of study counts is illustrated in Figure 2. Based on hand-searching citations of included studies, an additional 84 studies were included. After removing duplicates, we then conducted a title-abstract screening process, which excluded 2917 studies. We identified 89 studies for full-text screening. This number was further reduced to 21, which met our inclusion criteria.

Studies excluded after the full-text screen were found to be studies which: (1) are duplicates missed during the initial deduplication (n=2); (2) are withdrawn from publication (n=1); (3) have no accessible full-text (n=4); (4) propose AI in the conduct of continuous surgery, dosage, and treatment monitoring without human-mediated interpretation (n=4); (5) evaluate proprietary models (n=1); (6) suggest a CDSS model updating method (n=3); (7) evaluate CDSS models using only traditional discrimination metrics (n=18); (8) discuss clustering or population segmentation models (n=2); (9) are not about CDSS models or systems (n=9); (10) lack clinical workflow simulation (n=20); (11) propose synthetic data generation methods (n=1); (12) do not quantify potential impact (n=2); and (13) relate to *in vivo* evaluation (n=1).

Although the search did not restrict the publication year, all included studies were published from 2013 onward, with most (17/21, 81%) published between 2019 and 2023 (Multimedia Appendix 2). The majority of studies were conducted in the United States (n=15), followed by the United Kingdom (n=2), South Korea (n=1), Taiwan (n=1), and 2 cross-national collaborations (United States-France and United States-Italy). Study characteristics, including CDSS objectives, clinical domains, ISE strategies, simulation paradigms, and simulation scope, are summarized in Table 3. These are discussed in detail in the succeeding sections.

**Table 3.** Studies sorted by recency of publication along with their respective research locations, clinical domains, clinical decision support system (CDSS) objectives and classifications, *in silico* evaluation (ISE) strategies, and the simulation modeling paradigms implemented.

Study authors	Location	CDSS clinical domain	CDSS objectives and classification	ISE strategy	Simulation paradigm	Simulation scope
Rogers et al, 2023 [62]	The United States	Infectious diseases	Sepsis onset risk Prognostic	SBO^a^	Decision threshold cost sensitivity analysis	Cost-based optimization of a sepsis prediction model threshold
Thompson et al, 2023 [63,64]^b^	The United States	Radiology	Lung disease risk Triage	OCSA^c^	Markov State-transition models, individual-based	Radiologist image-reading workflow for disease diagnosis
Wornow et al, 2023 [42]	The United States	Cardiology	Peripheral arterial disease risk Diagnostic (screening)	OCSA	Discrete-event simulation with decision curve analysis	Clinic care delivery workflow from a patient visiting the clinic and the consequent treatment decision (ie, untreated, surgery, and medication) and multiple resource constraints within the workflow
Allen et al, 2022 [65]	The United Kingdom	ED^d^	Thrombolysis use Prescriptive	OCSA	Discrete-event simulation (NR^e^)	Stroke care delivery workflow from stroke onset, arrival, CT^f^ scan, up until thrombolysis administration.
Doubleday et al, 2022 [66]	The United States	None reported, domain-agnostic	Optimal treatment Prescriptive	SBO	Risk-adjusted decision tree (or random forest) optimization Resemblance to dynamic treatment regimes optimization	Optimization of individualized treatment rules in any treatment optimization scenario where historical data for efficacy and risk associated with past treatments are available
Rodriguez et al, 2022 [67]	The United States	Organ transplant	Mortality risk Prognostic	OCSA	State-transition models (microsimulation)	Patient longitudinal health states (ie, survival) simulation and single-resource constraint evaluation (ie, organs available for transplant)
Tardini et al, 2022 [53]	The United States and Italy	Oncology	Survival and toxicity incidence Prescriptive and prognostic	SBO	State-transition models (NR) Reinforcement learning for optimization	Cancer patient treatment stages, that is, from pretreatment features, initial chemotherapy decision, subsequent concurrent chemotherapy or radiotherapy decision, and finally the neck dissection decision.
Ziegelmayer et al, 2022 [68]	Germany	Oncology	Lung cancer screening Diagnostic (screening)	OCSA	Markov State-transition models (cohort-based simulation)	Cohort level “cost-effectiveness of an AI^g^-based system in the context of baseline lung cancer screening.”
Kamalzadeh et al., 2021 [69]	The United States	Endocrinology	Diabetes risk Diagnostic (screening)	SBO	State-transition models: various Markov models Optimization of partially observable Markov decision process	Patient longitudinal health states (ie, diabetes status) simulation and single-resource constraint evaluation (ie, screening capacity)
Kim et al, 2021 [43]	South Korea	Infectious diseases	Maximum severity level Prognostic and triage	OCSA	Discrete-event simulation	Patients with COVID-19 triage based on severity (ie, assignment to ICU^h^ and general ward)
Misic et al, 2021 [70]	The United States	ED	Readmission risk Prognostic	OCSA	Discrete-event simulation (NR)	Postoperative care pathway until discharge or readmission decision
Tang et al, 2021 [71]	The United States	Oncology	Optimal treatment Prescriptive	SBO	Multistage decision-making Reinforcement learning for optimization	Two-stage dynamic treatment regime with each stage involving a test decision and subsequent treat decision steps.
Alenany and Cadi, 2020 [72]	The United States and France	ED	Readmission risk Triage	OCSA	Discrete-event simulation (NR)	From emergency department (theoretical) arrival until departure from ED: workflow states and transitions
D’Andrea et al, 2020 [73]	The United States	Oncology	Lung cancer status Diagnostic	OCSA	Discrete-event simulation	Complete diagnostic and surveillance pathway Explores 3 different pathway scenarios
Allen et al., 2019 [74]	The United Kingdom	ED	Thrombolysis use Prescriptive	OCSA	Discrete-event simulation (NR)	Stroke care delivery workflow from stroke onset, arrival, CT scan, up until thrombolysis administration.
Hunter-Zinck et al, 2019 [75]	The United States	ED	Doctors’ orders Prescriptive	OC^i^	Discrete-event simulation (NR) Time-to-event data	From ED (theoretical) arrival until departure from ED: Simulation of LOS^j^ based on aggregate time-interval across orders, triage, and disposition
Tsai et al, 2019 [76]	Taiwan	Intensive care	Extubation failure Prognostic	OCSA	Bayesian decision tree analysis	Bayesian decision analysis of costs (ie, ICU days) attributed to a decision to extubate. Sensitivity analysis was conducted.
Hager et al, 2018 [77]	The United States	Oncology	Survival and adverse events Prescriptive	SBO	Dynamic treatment regimes optimization	Simulation of the DTR^k^ optimization formulation was done using synthetic and real world data (North American Leukemia Intergroup Study C9710)
Lee et al, 2015 (“Applying...") [78]	The United States	Oncology	Optimal screening policy Diagnostic (screening)	SBO	Discrete-event simulation Reinforcement learning for optimization	Screening pathway for hepatocellular carcinoma
Lee et al, 2015 (“Transforming...") [79]	The United States	ED	Readmission risk Prognostic	SBO	Agent-based modeling	Actual emergency department workflow process for a partner care institution.
Ashour and Kremer, 2013 [80]	The United States	ED	Triage Triage	OC	Discrete-event simulation	ED (theoretical) triage until (1) immediate treatment or (2) sending the patient to the waiting room

a SBO: simulation-based optimization.

b A related study by Thompson et al [81] was published while analysis was underway; however, we only included their previous works found within the search period.

c OCSA: outcome comparison with sensitivity analysis.

d ED: emergency department.

e NR: not explicitly reported but classified by the study team as such.

f CT: computed tomography.

g AI: artificial intelligence.

h ICU: intensive care unit.

i OC: outcome comparison.

j LOS: length of stay.

k DTR: dynamic treatment regimes.

### RQ1: What Clinical Domain and Decision-Making Task Does the CDSS Aim to Assist?

CDSS decision-making objectives across the included studies are found to align with 4 commonly described CDSS task categories: diagnostic and screening, prognostication, triaging, and prescriptive decision support. These task classifications are used to characterize the intended CDSS function across the reviewed literature.

The included papers encompassed various clinical domains, most prominently the emergency department (n=7), oncology (n=6), and infectious diseases (n=2), as well as organ transplantation, endocrine disorders, radiology, and intensive care. This distribution highlights the breadth of CDSS applications across diverse specializations. Additional details of CDSS aims and domain-specific applications are provided in Table 3 and Multimedia Appendix 3.

### RQ2: What Outcome Measures are Used to Evaluate Potential Impact?

Outcome measures reported across the included studies are mapped to the four *a priori* thematic categories: patient-related, cost-related, provider-related, and process-related. Patient-, cost-, and provider-related outcomes are common themes in the CDSS literature. Additionally, we coded process-related outcomes that reflect how CDSS interventions may influence operational efficiency within clinical pathways. Examples of each outcome group are detailed in Table 4.

**Table 4.** Definitions and examples of outcomes and parameters used in *in silico* evaluation (ISE) studies of clinical decision support systems (CDSSs).

Theme	Definition	Example parameters	Example outcomes
Patient	States or attributes specific to patients that influence or result from simulation logic	Age, comorbidities, biomarker levels, disease stage, and risk scores	Survival, disease, progression, adverse events, and treatment response
Process	Elements of the care pathway, workflow, resource flow, or timing of care events	Triage time, wait times, length of stay, and time-to-diagnosis	Throughput, delays avoided, and bottleneck reduction
Cost	Monetary or economic values used to parameterize resource consumption or compare outcomes	Costs of test and treat decisions	Cost-effectiveness, cost savings, and incremental cost per quality-adjusted life year
Provider	Factors related to clinician behavior, availability, or interaction with CDSS.	Provider availability, adherence to CDSS, and override rates	Not found in the review; possible examples are measures of provider burnout or well-being

Out of the 21 included studies, 90% (n=19) evaluated CDSS using patient-outcome measures. Specifically, studies investigated clinical events such as undetected cancer cases [73], micro- and macrovascular events prevented [69], early-stage cancer detection [78], and outcomes in large-vessel occlusion stroke [63,64]. Other patient outcomes included mortality [43,53,67,69,73], restricted survival time [77], quality-adjusted life-years [42,69,73], readmissions [70], treatment efficacy [66,71], treatment use [65,74], and length of stay [72,75,76,79,80].

Outcome measures focused on reducing health care costs underscored the need for financially sustainable health care CDSS interventions. Cost-effectiveness outcomes were considered by 29% (n=6) of studies. The specific outcomes were incremental cost-effectiveness ratios [68,69,73], cost savings from anticipating clinical events [70,78], cost reductions from proactive care [62], and aggregate incurred costs [75]. Nearly half of the included studies (n=10) also used outcomes related to health service delivery management, such as resource allocation [65,73,74], elapsed time [64,69,72], referral rates [67], clinical workflow throughput [70,79,80], and procedure usage [65]; these are classified in our work as process-related outcomes.

Several studies combined multiple outcome measures, including those that consider both process and patient outcomes (n=7, 33%), evaluating the impact of CDSS on patient well-being through the lens of process efficiency and optimization. Other studies (n=3, 14%) combine patient outcomes with cost-effectiveness measures. Notably, a few studies (n=3, 14%) conducted a more holistic analysis that combined patient outcomes, process outcomes, and cost-effectiveness. None were found to have conducted ISE to measure provider-related outcomes [82,83]. A nonexhaustive list of outcomes and parameters used in ISE studies is presented in Table 4, organized by patient, process, cost, and provider-related themes.

### RQ3: What Clinical Workflow or Pathway Parameters are Simulated by the Study?

Simulation parameters identified across the included studies were grouped into the same 4 *a priori* thematic categories used for outcome measures: patient, process, cost, and provider. This reflects the aspects of the care delivery system that each parameter represents.

More than 57% (n=12) of studies conducted simulations that incorporated both patient- and process-related parameters. Fewer studies (n=3, 14%) conducted simulations that integrated 3 of the 4 simulation parameter classifications: patient, cost, and process-related parameters [69,73,79]. None considered patient, cost, process, and provider-related parameters simultaneously. The simulation parameters [56] used to design simulations that mimic real-world clinical workflow scenarios were either estimated from prior literature, clinical experience [73,76], retrospective observational data [42,69], or clinical trial data [73]. A detailed account of the use of patient, provider, cost, and process parameters, along with the respective outcomes measured by each included study, is presented in Table 5.

**Table 5.** Outcome measures and parameters used by the included studies. Studies are sorted according to the recency of publication.

*In silico* evaluation component (study authors)	Outcomes^a^				Simulation parameters^b^			
	Provider	Patient	Cost	Process	Provider	Patient	Cost	Process
Rogers et al, 2023 [62]			✓		✓		✓	✓
Thompson et al, 2023 [63,64]		✓		✓	✓			✓
Wornow et al, 2023 [42]		✓			✓	✓		✓
Allen et al, 2022 [65]		✓		✓		✓		✓
Doubleday et al, 2022 [66]		✓				✓		✓
Rodriguez et al, 2022 [67]		✓		✓		✓		✓
Tardini et al, 2022 [53]		✓				✓		✓
Ziegelmayer et al, 2022 [68]			✓			✓		✓
Kamalzadeh et al, 2021 [69]		✓	✓	✓		✓	✓	✓
Kim et al, 2021 [43]		✓				✓		✓
Mišić et al, 2021 [70]		✓	✓	✓	✓	✓		
Tang et al, 2021 [71]		✓				✓		✓
Alenany and Cadi, 2020 [72]		✓		✓		✓		✓
D’Andrea et al, 2020 [73]		✓	✓	✓		✓	✓	✓
Allen et al, 2019 [74]		✓		✓		✓		✓
Hunter-Zinck et al, 2019 [75]		✓	✓			✓	✓	✓
Tsai et al, 2019 [76]		✓	✓			✓		✓
Hager et al, 2018 [77]		✓				✓		✓
Lee et al, 2015 (“Applying….”) [78]		✓	✓		✓	✓		✓
Lee et al, 2015 (“Transforming…”) [79]		✓		✓		✓	✓	✓
Ashour and Kremer, 2013 [80]		✓		✓		✓		✓

a Outcomes can be either provider-, patient-, cost-, or process-related.

b Simulation parameters are the operational details of the simulated clinical workflow and are classified as provider-, patient-, cost-, or process-related.

Patient-related parameters have accounted for health-related factors, such as cancer staging [73], utilities attributed to false-positive or false-negative diagnoses [68], chronic disease severity [69], recovery time (eg, observed time spent in the ICU) [76], and aggregate cohort characteristics [78]. There are also other patient-related parameters, such as patient preferences for undergoing an examination [71] and health insurance coverage [79].

Process- or workflow-related parameters have frequently been used to define transitions between discrete states or events [65,72,74,79]. These parameters can be related to structured treatment regimens or plans [53,71,77], risk levels [66], resource availability [70], and resource allocation policies [43,63,64,67]. In addition, some studies use the predictive CDSS’s sensitivity, specificity, and decision thresholds as process parameters in potential impact assessments [42,43,62,64,69,73]. While no studies evaluated the CDSS’s potential impact on provider *outcomes*, several studies (n=5, 24%) used provider-related *parameters*. Provider-related parameters include provider effectiveness [70], serviceable capacity [42], the probability that providers view a CDSS-generated alert and subsequently adhere to it [42,62], and provider reading rates [63,64]. Cost-related parameter values include attributes related to workflows, clinical procedures, or order sets [68,73,75]; clinical events; patient-related costs [69]; costs attributed to false alarms and missed cases [62]; and costs attributed to patient reimbursement characteristics [80].

### RQ4: What Simulation Modeling Paradigm is Used?

ISPOR reports on various paradigms in simulation modeling [28,55,57,84]. One of these is the family of dynamic simulation models [28]. This family includes system dynamics, discrete-event, and agent-based modeling. Discrete event simulation is used in 29% (n=6) of clinical workflow simulations [42,43,73,78-80]. One study reported the use of both discrete-event simulation and agent-based modeling to simulate multiple emergency department units within a single institution [79]. Another study proposed a domain-agnostic discrete-event simulation framework to accommodate a variety of clinical workflows; however, only the peripheral artery disease case was discussed [42].

A smaller fraction of studies (n=4, 19%) reported the use of state-transition models [57]. Kamalzadeh et al [69] optimized decision-making using a partially observable Markov decision process. Rodriguez et al [67] reported a microsimulation for a per-patient transition between states. Thompson et al [63,64] used a Markov model to evaluate an AI-assisted triage process for radiological images. Ziegelmayer et al [68] reported a cohort-based Markov model of AI-supported computed tomography, considering cost-effectiveness and the impact of the CDSS model’s performance on outcomes.

Finally, among those reviewed are studies that model the hierarchical decision-making process inherent in test-and-treat scenarios. Studies (n=3, 14%) have used dynamic treatment regime optimization to determine optimal sequential treatment strategies based on dynamic patient states throughout the care pathway. Tang et al [71] and Tardini et al [53] further combined reinforcement learning with dynamic treatment regime optimization to maximize reward functions based on treatment efficacy indicators (ie, prostate-specific antigen) and survival outcomes, respectively. Hager et al [77] proposed a dynamic treatment regime optimization study specific to censored survival data. Studies have also discussed the development of a decision tree or random forest to predict treatment efficacy, with risk scores adjusted during the CDSS model’s derivation [66,76].

## Discussion

### Overview

CDSS are intended to enhance patient safety, improve the quality of care, and support health care providers in making informed clinical decisions. Traditional CDSS relied on care standards, rule-based scoring systems, or clinical protocols [1,2]. More recent AI- and ML-based CDSS are developed from data-driven algorithms, which are susceptible to drift in data distributions and changes in clinical practice. These CDSS require periodic evaluation and typically need integration with existing health care IT infrastructures to maximize their value while ensuring reliability over time. These characteristics have led to a growing interest in ISE methods, which enable the assessment of potential impact before implementation, facilitate timely planning for emergent scenarios (eg, pandemics), and potentially monitor performance drift after implementation [42,70].

### Screening Yield, Timeline of Studies, and Their Geographical Distribution

Less than 1% of screened literature (21/3307) met our inclusion criteria. This is amid numerous studies (886/3307) that propose a CDSS between 1994 and 2023. Among the 886 CDSS studies, only 3 % extend evaluation to include ISE (ie, clinical workflow simulations) to measure potential impacts on patient, process, and cost outcomes. This highlights a gap in the uptake of ISE methods.

An increase in ISE studies was observed beginning around 2019 (Multimedia Appendix 2), which we attribute to the convergence of global health system pressures and rapid advances in AI. The COVID-19 pandemic exposed mismatches between surging clinical demand and limited resources, heightening the need for methods that can anticipate workflow bottlenecks and evaluate CDSS under constrained, rapidly changing conditions [85-89]. At the same time, accelerating developments in AI, particularly the widespread adoption of deep learning and early generative AI systems [90,91], led to a proliferation of CDSS models and intensified concerns about their real-world impact and safety. We posit that these forces together created strong incentives for simulation-based approaches, such as ISE, which offer a scalable, low-risk means of evaluating CDSS prior to deployment.

No studies are included that demonstrate ISE use in low- to middle-income countries (LMICs). This presents a missed opportunity, especially since LMICs face precisely the kinds of operational constraints for which workflow-sensitive evaluation could be most informative [92,93]. Given our team’s health services research focus in Southeast Asia, future work is well-positioned to address this gap by developing and evaluating CDSS using ISE methodologies tailored to the realities of LMIC health systems.

### ISE in Various CDSS Domains

Our review observed that the majority of the included ISE studies proposed CDSS to aid emergency (n=7) and oncology (n=6) care pathways. These 2 domains share several characteristics that make system-level evaluation crucial: both involve complex, multistep care processes, time-sensitive decision-making, constrained resources, and high variability in patient trajectories. In emergency care, treatment delays, crowding, and capacity limitations can substantially affect the realized benefit of a CDSS, even when it exhibits strong predictive performance. Similarly, oncology pathways involve sequential treatment and risk-stratification decisions, the effectiveness of which can be substantially reduced by downstream bottlenecks. ISE provides a mechanism to anticipate whether proposed CDSS interventions would translate into meaningful improvements when considering the broader pathway.

We identified four categories of aims: (1) screening and diagnosis, (2) prognostication, (3) prescriptive, and (4) triaging. Common aims discussed in CDSS reporting guidelines are diagnosis and prognostication. Cowley et al [5] distinguish prescriptive aims as recommendations for effective treatment. We included triage as a distinct aim classification to distinguish it from diagnosis-related decisions that occur later in the emergency care pathway. Unlike the other aims, triage-oriented CDSS explicitly influence patient flow, resource prioritization, and care-escalation decisions despite relatively minimal information about patients’ conditions and histories. This category is particularly relevant for ISE, as the consequences of these decisions on waiting times, capacity bottlenecks, and care delivery sequencing cannot be measured using traditional metrics.

### ISE Outcomes and Simulation Parameters

**Figure 3.**
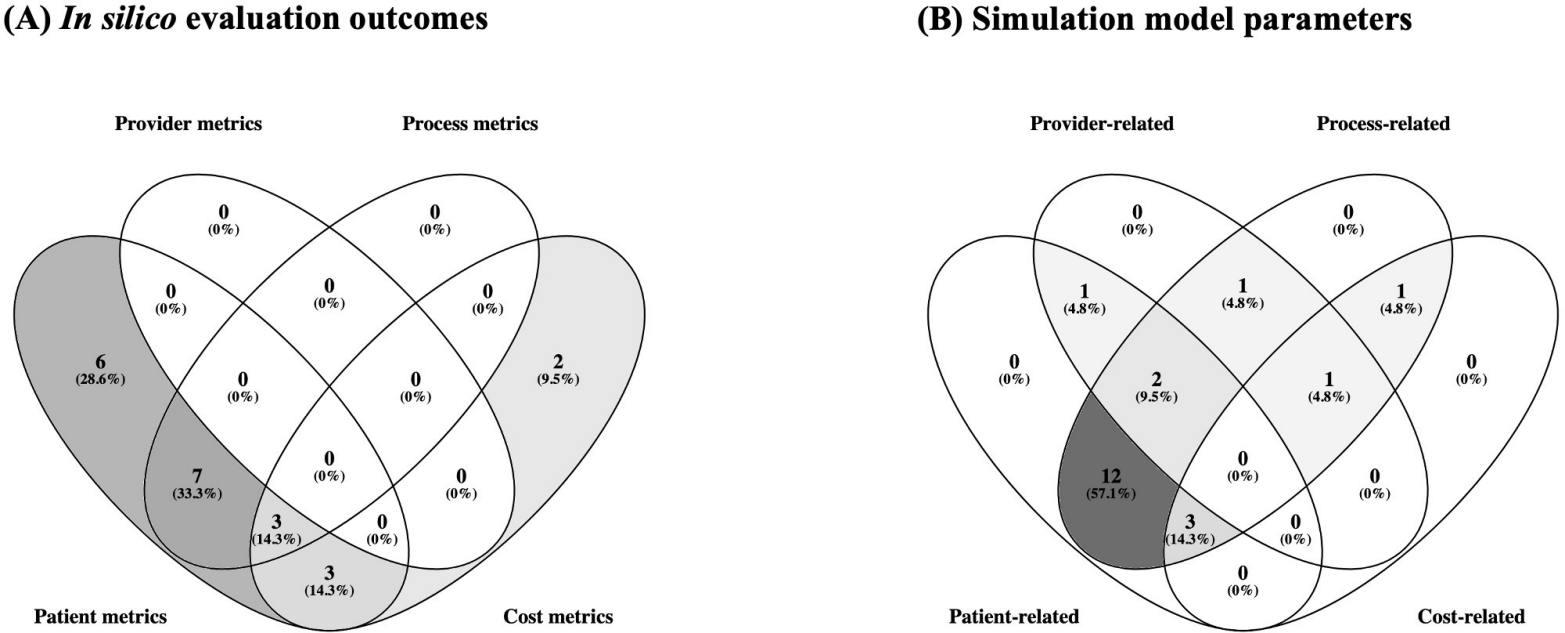
Venn diagrams [94] show the distribution of studies across themes identified in (**A**) *in silico* evaluation metrics and (**B**) simulation model parameters considered by the proposed *in silico* evaluation frameworks.

ISE enables the simultaneous evaluation of multiple, potentially conflicting outcomes while accounting for various system parameters. Figure 3 illustrates the distribution of outcomes and parameter themes, thereby highlighting the simultaneous consideration of these themes across the reviewed studies.

The majority of the reviewed ISE studies combine patient-related outcomes with process-related outcomes (Figure 3). This observation contributes to the CDSS literature, which often excludes clinical workflow (ie, process-related) factors as evaluation outcomes of interest [7,18,23,59]. The observation also supports the value of ISE methods in expanding the scope of evaluation to consider operational bottlenecks, efficiency, and resource constraints.

ISE studies that assess patient and cost outcomes align with longstanding health care aims to prioritize patient well-being and cost-effectiveness, respectively [95]. In addition, process outcomes support the third aim, population health, by ensuring that quality and cost-effective care is efficiently distributed to the population. Specific process outcomes included throughput, referral rates, and treatment yield, all of which shed light on care coverage and efficiency.

A critical gap in current ISE applications is the absence of provider-related outcome evaluation, despite providers’ key role in workflow dynamics (Figure 3A). A few studies (n=5), however, have demonstrated that simulation models are inherently capable of incorporating such considerations [42,62-64,70]. They address this gap by incorporating provider characteristics as simulation parameters (Figure 3B), such as provider availability, CDSS adherence, and decision override rates, thereby acknowledging providers as critical elements of the simulated care pathway. This direction toward considering provider factors as simulation outcomes and parameters is consistent with calls to expand health care aims to include care provider well-being, such as in the quadruple aims [95-97].

To facilitate provider-outcome measurement, these parameters can then be linked to modeled outcomes such as queue lengths, overtime hours, workload burden, or the probability of missed or ignored alerts. For example, CDSS-triggered recommendations could be encoded to increase task volume or interrupt workflow, allowing simulations to quantify how different alert thresholds or workflow designs propagate into provider workload or burnout risk. Similarly, varying staffing patterns or shift lengths within discrete event or agent-based models could help evaluate whether CDSS-driven changes in patient flow exacerbate provider strain.

By formalizing these constructs as model inputs and outputs, future ISE studies could extend the evaluation of CDSS beyond patient, process, and cost outcomes to include provider well-being, thereby aligning with the global shift to include such measures in the quadruple aims of health care [95-97].

### ISE Objectives, Strategies, and Simulation Modeling Paradigms

We also grouped studies by higher-level motivations for using ISE. The different classifications of ISE objectives can be defined as follows: *the outcome comparison* group, which conducts straightforward comparisons of clinical usefulness for CDSS implementation without sensitivity analysis or simulation-based optimization; *the outcome comparison with sensitivity analysis* group, which analyzes the sensitivities of outcome measures to various workflow parameters and scenarios; and, finally, *the simulation-based optimization* group, which integrates workflow simulation parameters into CDSS model development.

**Figure 4.**
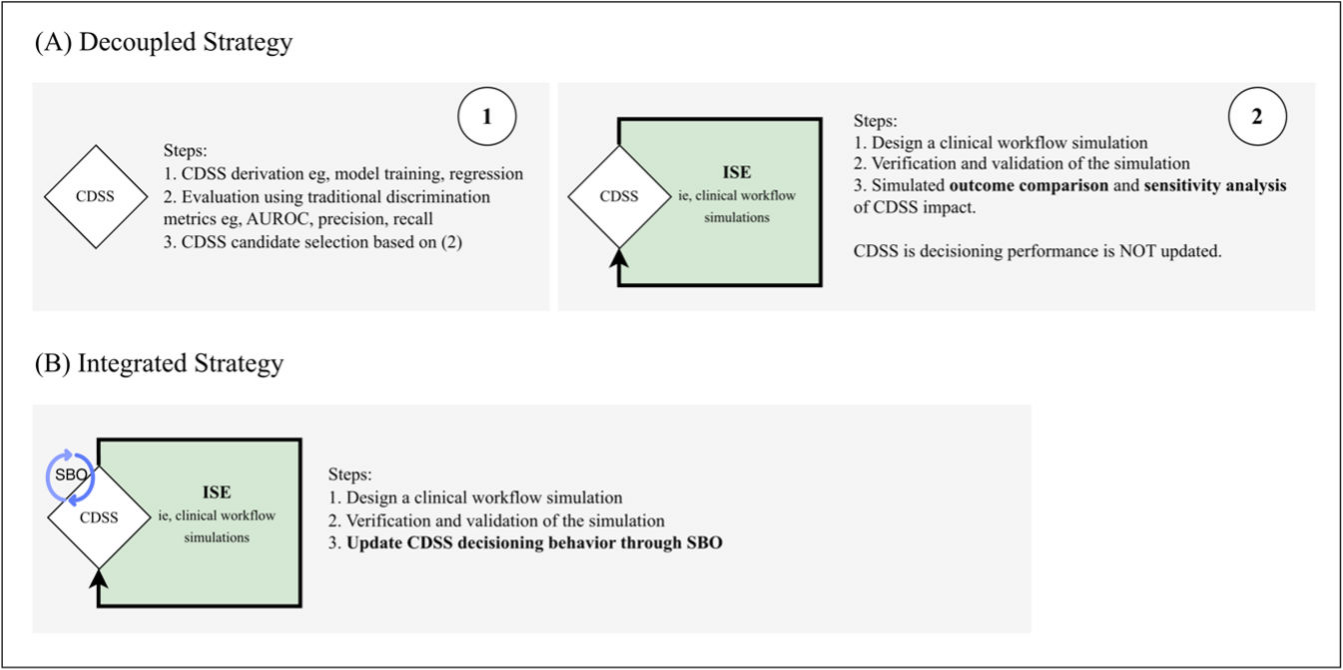
Distinct strategies underpinning clinical workflow simulations for *in silico* evaluation (ISE) of clinical decision support systems (CDSSs). (A) A decoupled ISE strategy develops or trains CDSS independent of a simulated care pathway and subsequently evaluates the CDSS (without updating) using ISE. (B) An integrated strategy optimizes CDSS within a simulated workflow through simulation-based optimization strategies. AUROC: area under the receiver operating characteristic; SBO: simulation-based optimization.

From the observed specific evaluation objectives, two distinct families of strategies underpinning the workflow simulation have emerged: (1) *decoupled* (ie, outcome comparison with and without sensitivity analysis), and (2) *integrated* (ie, simulation-based optimization) strategies. This distinction provides a conceptual framework for structuring future ISE studies. Both strategies are illustrated schematically in Figure 4.

In the decoupled strategy, CDSS models are first developed and optimized based on localized discrimination metrics. The CDSS is then embedded in a simulation model, often as a decision node, within a virtual care pathway [42,63,64]. The decoupling is valuable for isolating how workflow constraints shape the realized impact of an otherwise well-performing model. During pre-implementation evaluation, the strategy allows developers or implementers to stress-test CDSS performance across plausible clinical scenarios without modifying the underlying algorithm. This enables the parallel resolution of mismatches between model predictions and system capacity, which are otherwise not evident in non-ISE methods.

In the integrated strategy, clinical workflows are simulated to update or optimize CDSS performance through simulation-based optimization. Specific methods for implementing the integrated strategy include agent-based modeling [79], dynamic treatment regime optimization [77], dynamic treatment regime optimization with reinforcement learning [53,71], reinforcement learning with discrete event simulation [78], estimation of risk-adjusted decision trees and random forests [66], partially observable Markov decision processes [69], and cost-minimization via decision curve analysis [62].

In earlier simulation methods, pathway outcomes are inherently factored into model development; for example, as reward functions in reinforcement learning studies [71,78], as optimization objectives in dynamic treatment regime optimization [53,71,77], and as model estimation criteria in risk-adjusted decision tree treatment efficacy predictors [66]. An integrated strategy, therefore, provides a mechanism for discovering context-optimized CDSS configurations that balance predictive accuracy with operational feasibility.

Assessing the potential impact of a CDSS via ISE [30,56] requires simulation models that can accommodate the stochastic and dynamic nature of clinical workflows and systems. Stochasticity may stem from probabilistic decision-making through CDSS, provider and patient preferences, and fluctuating resource constraints. Workflows are also dynamic due to factors such as disease progression, health deterioration or improvement, and time-dependent health care supply-and-demand structures.

Among the specific simulation paradigms that capture the stochastic and dynamic characteristics of workflows are dynamic simulation models, particularly discrete-event simulation and agent-based modeling [28,42,43,73,78-80]. A subset of studies also used dynamic treatment regime optimization approaches with reinforcement learning to optimize clinical decisions based on domain-informed reward functions, such as biomarker responses or survival outcomes [53,71]. These approaches also reflect a growing trend within this subset of studies toward embedding dynamic, personalized, and data-driven logic into the simulation-based optimization and evaluation of CDSS.

The paradigms identified are well-established in non–health care operations research and are widely adopted in health services research [55,84]. Consolidated guidance on the health care applications of dynamic simulations [28,36] and state-transition modeling [57] has existed since 2015 through ISPOR initiatives. However, the specific use of such methods for CDSS evaluation, that is, ISE, remains critically under-explored. This gap limits CDSS stakeholders' ability to anticipate workflow consequences. Expanding the application of ISE, therefore, represents a timely and necessary step toward more reliable, context-aware evaluation of CDSS.

### Comparison With Prior Work

Prior work on CDSS evaluation has largely focused either on narrow, model-level assessments conducted before deployment or on broader, but substantially more resource-intensive, postdeployment evaluations and trial-based studies. In contrast, our review positions ISE as an intermediary approach: one that enables the assessment of outcomes relevant to the broader clinical care pathway while avoiding the resource demands associated with live implementation or clinical trials.

Several studies have investigated the evaluation of CDSS effectiveness using traditional trial-based impact assessments, such as randomized controlled trials and postdeployment evaluation studies. They highlighted provider adherence and behavior change [61], real-world usage rates [98], and other impacts on care providers’ performance [99]. These aspects may also be captured through simulated care pathways without requiring pilot or full-scale implementation, as emphasized in our scoping review.

Other studies have highlighted process-related factors that are evaluated when measuring CDSS impact postdeployment [61,100]. A meta-analysis of 10 randomized controlled trials also found that CDSS interventions yielded modest improvements in care process adherence, while having minimal effects on patient outcomes [100]. This highlights a persistent gap between the CDSS promise and real-world impact. Our work addresses this gap by positioning ISE as a means to assess these performance limitations and the necessary design improvements before significant resource allocation.

Several recent CDSS development frameworks and methodological guidelines have influenced our approach. The majority focused on evaluating CDSS [5,24,25]. One study broadly covered the CDSS development and evaluation lifecycle, from derivation and validation to impact assessment and long-term implementation [2,3,5]. Others extended this to tackle CDSS updating and maintenance [3,29]. Specific guidance for early-stage evaluation of AI-enabled CDSS, emphasizing usability, iterative updates, and structured reporting, was also proposed [38,39]. A recurring stage in at least 2 proposed frameworks is the “silent” [2,39] or “shadow-mode” [38] evaluation, which aligns with our focus on increasing evidence of value prior to substantial resource allocation. However, these suggestions already assume an integration with the needed data sources, albeit minimally sufficient, to operate in parallel with usual care practice. Across the abovementioned related literature, the use of ISE remained unaddressed.

Collectively, the existing literature underscores the importance of stronger preclinical evaluation mechanisms, broader post-deployment outcomes evaluation, and standard reporting and methodological guidelines for CDSS development. However, none of the studies cited systematically reviewed the extent to which ISE has been used to evaluate CDSS.

### Limitations and Future Work

First, as is typical of scoping reviews, our objective is to map and describe the existing landscape without critically appraising its quality or conducting a meta-analysis. This approach enables a broader view of the scope and gaps and is consistent with scoping review methodology.

Second, this review is limited by the exclusion of non-English-language journals and preprints. This is a necessary and pragmatic limitation given our research team’s inability to reliably interpret and critically synthesize non-English academic literature.

Third, for ISEs to capture actual clinical care pathways, information that allows for simulation model verification and validation should be available. Investigating the critical aspects of data availability is beyond the intended scope of our review. Nonetheless, we recognize that future research should adopt more concrete strategies, such as benchmarking simulation models against retrospective electronic health record data, stress-testing assumptions across diverse care scenarios, domain-expert validation of simulation behavior against experience, and conducting sensitivity analyses under real-world workflow constraints. We refer the reader to an ISPOR report for more detailed guidelines on simulation model verification and validation [54]. Furthermore, future work could investigate pragmatic considerations for the use of ISEs in the CDSS development lifecycle.

Finally, our review focuses on the methodological applications of ISE. While a comprehensive ethical or regulatory analysis is beyond the scope of this study, we echo prior calls for robust governance frameworks to guide the responsible development, evaluation, and deployment of CDSS [30,54]. Future work is needed to bridge methodological advances in ISE with ethical, regulatory, and accountability considerations.

### Conclusions

As CDSS evolve from rule-based logic to data-driven algorithms, the need for context-aware, flexible, and scalable evaluation strategies is becoming increasingly critical. Our review revealed that ISE remains underused despite evidence of its strong potential for the pre-implementation assessment of CDSS. The growing body of research in the development of algorithm-based CDSS calls for a shift in how evidence for their safety and reliability is generated. Our review identified a subset of studies that propose more robust approaches for evaluating CDSS using simulated clinical workflows. We identified common themes among the simulation parameters and the outcomes being evaluated. Additionally, we identified common stochastic and dynamic simulation methods proposed and how they are integrated with existing CDSSs.

## Supplementary material

10.2196/72472 Multimedia Appendix 1 Keywords used and other details of the literature search, including a sample encoding sheet that uses Notion.so.

10.2196/72472 Multimedia Appendix 2 Timeline of studies included in the scoping review.

10.2196/72472 Multimedia Appendix 3 Summary of information extracted from included articles during the scoping review.

10.2196/72472 Checklist 1 PRISMA-ScR checklist.

## Abbreviations

AI: artificial intelligence
CDSS: clinical decision support system
ISE: *in silico* evaluation
ISPOR: Professional Society for Health Economics and Outcomes Research (formerly the International Society for Pharmacoeconomics and Outcomes Research)
LMIC: low- to middle-income country
ML: machine learning
PRISMA-ScR: Preferred Reporting Items for Systematic Reviews and Meta-Analyses Extension for Scoping Reviews
RQ: research question
