# Supplementary material for: In Silico Evaluation of Algorithm-Based Clinical Decision Support Systems Based on Care Pathway Simulation Models: Scoping Review
Source: JMIR AI. 2026 Mar 24;5:e72472. doi: 10.2196/72472 (PMC13012236; doi:10.2196/72472)
Supplement: Multimedia Appendix 1 [file ai-v5-e72472-s001.docx]

# Supplementary Material

## Keywords and details of the search

**Table S1.** Programmatic database search details. Only "English" articles were included.

| **Keywords** | | **Search strategy** | |
| --- | --- | --- | --- |
| CINAHL, Search date: 19/05/2023 | | | |
| Concept: CDS models, algorithms, and systems | | |  |
| Strings | “machine learning” OR “deep learning” OR “artificial intelligence” OR “reinforcement learning” OR “supervised learning” OR “unsupervised learning” OR “supervised machine learning” OR “unsupervised machine learning” OR “semi- supervised machine learning” OR “self- supervised machine learning” OR “semi- supervised learning” OR “self-supervised learning” OR “expert system” | | Title, abstract |
| MeSH | (MH "Artificial Intelligence+") OR (MH "Expert Systems") | |  |
| Concept: Objective of the CDS model | | |  |
| Strings | "clinical decision support" OR "clinical decision making" OR "diagnosis" OR "prognosis" OR "diagnoses" OR "prognoses" OR "screening" OR "triage | | Title, abstract |
| MeSH | (MH "Decision Support Systems, Clinical") OR (MH "Triage") OR (MH "Diagnosis+") OR (MH "Prognosis+") | |  |
| Concept: Evaluation objective | | |  |
| Strings | "credibility" OR "validation" OR "verification" OR "potential impact" OR "impact assessment" OR "decision analysis" OR "decision analytic measure*" OR "decision analytic model*" OR "net benefit" OR "decision curve*" OR "calibration" OR "model tuning" OR "credibility" OR "cost benefit analysis" OR "cost utility analysis" OR "cost-benefit analysis" OR "cost-utility analysis" OR "cost and benefit" OR "cost and utility" OR "model revision" OR "usefulness" OR "performance" OR "utility" | | Title, abstract |
| MeSH | (MH "Decision Making, Patient+") OR (MH "Decision Making, Clinical+") OR (MH "Decision Support Techniques+") OR (MH "Systems Validation") OR (MH "Health Impact Assessment") OR (MH "Cost Benefit Analysis") OR (MH "Costs and Cost Analysis+") OR (MH "Health Care Costs+") OR (MH "Outcomes (Health Care)+") | |  |
| Concept: Evaluation strategy | | |  |
| Strings | "in silico" OR "computer simulation" OR "simulation" OR "digital twin" OR "digital evaluation" OR "digital simulation" OR "computational simulation" OR "computational evaluation" OR "computational model" OR "pre-implementation" OR "pre-deployment" | | Title, abstract |
| MeSH | 'computer simulation'/exp OR 'digital twin'/exp OR 'computer model'/exp | |  |
| Embase, Search date: 19/05/2023 | | | |
| Concept: CDS models, algorithms, and systems | |  | |
| Strings | 'machine learning':ti,ab,kw OR 'deep learning':ti,ab,kw OR 'artificial intelligence':ti,ab,kw OR 'reinforcement learning':ti,ab,kw OR 'supervised learning':ti,ab,kw OR 'unsupervised learning':ti,ab,kw OR 'supervised machine learning':ti,ab,kw OR 'unsupervised machine learning':ti,ab,kw OR 'semi-supervised machine learning':ti,ab,kw OR 'self-supervised machine learning':ti,ab,kw OR 'semi-supervised learning':ti,ab,kw OR 'self-supervised learning':ti,ab,kw OR 'expert system':ti,ab,kw | Title, abstract, keywords | |
| Emtree | 'machine learning'/exp OR 'self supervised learning'/exp OR 'expert system'/exp |  | |
| Concept: Objective of the CDS model | |  | |
| Strings | 'clinical decision support':ti,ab,kw OR 'clinical decision making':ti,ab,kw OR 'diagnosis':ti,ab,kw OR 'diagnoses':ti,ab,kw OR 'prognosis':ti,ab,kw OR 'prognoses':ti,ab,kw OR 'screening':ti,ab,kw OR 'triage':ti,ab,kw | Title, abstract, keywords | |
| Emtree | 'patient triage'/exp OR 'screening test'/exp OR 'prognosis'/exp OR 'diagnosis'/exp OR 'clinical decision support system'/exp OR 'clinical decision rule'/exp OR 'prognostic score'/exp |  | |
| Concept: Evaluation objective | |  | |
| Strings | 'validation':ti,ab,kw OR 'verification':ti,ab,kw OR 'potential impact':ti,ab,kw OR 'impact assessment':ti,ab,kw OR 'decision analysis':ti,ab,kw OR 'decision analytic measure':ti,ab,kw OR 'decision analytic model':ti,ab,kw OR 'net benefit':ti,ab,kw OR 'decision curve':ti,ab,kw OR 'decision curve analysis':ti,ab,kw OR 'calibration':ti,ab,kw OR 'model tuning':ti,ab,kw OR 'credibility':ti,ab,kw OR 'cost benefit analysis':ti,ab,kw OR 'cost utility analysis':ti,ab,kw OR 'cost-benefit analysis':ti,ab,kw OR 'cost-utility analysis':ti,ab,kw OR utility:ti,ab,kw OR 'model revision':ti,ab,kw OR 'usefulness':ti,ab,kw OR 'performance':ti,ab,kw | Title, abstract, keywords | |
| Emtree | 'decision making'/exp OR 'cost benefit analysis'/exp OR 'cost utility analysis'/exp |  | |
| Concept: Evaluation strategy | |  | |
| Strings | 'in silico':ti,ab,kw OR 'computer simulation':ti,ab,kw OR 'simulation':ti,ab,kw OR 'digital twin':ti,ab,kw OR 'digital evaluation':ti,ab,kw OR 'digital simulation':ti,ab,kw OR 'computational simulation':ti,ab,kw OR 'computational evaluation':ti,ab,kw OR 'computational model':ti,ab,kw OR 'pre-implementation':ti,ab,kw OR 'pre-deployment':ti,ab,kw | Title, abstract, keywords | |
| Emtree | 'computer simulation'/exp OR 'digital twin'/exp OR 'computer model'/exp |  | |
| Other filters | |  | |
| Article types | ('clinical trial'/de OR 'major clinical study'/de OR 'observational study'/de OR 'validation study'/de OR 'validation process'/de) |  | |
| PsycInfo, Search date: 19/05/2023 | | | |
| Concept: CDS models, algorithms, and systems | |  | |
| Strings | “machine learning” OR “deep learning” OR “artificial intelligence” OR “reinforcement learning” OR “supervised learning” OR “unsupervised learning” OR “supervised machine learning” OR “unsupervised machine learning” OR “semi- supervised machine learning” OR “self- supervised machine learning” OR “semi- supervised learning” OR “self-supervised learning” OR “expert system” | Title, abstract, keywords | |
| Concept: Objective of the CDS model | |  | |
| Strings | "clinical decision support" OR "clinical decision making" OR "diagnosis" OR "prognosis" OR "diagnoses" OR "prognoses" OR "screening" OR "triage" | Title, abstract, keywords | |
| Concept: Evaluation objective | |  | |
| Strings | "credibility" OR "validation" OR "verification" OR "potential impact" OR "impact assessment" OR "decision analysis" OR "decision analytic measure" OR "decision analytic model" OR "net benefit" OR "decision curve" OR "decision curve analysis" OR "calibration" OR "model tuning" OR "credibility" OR "cost benefit analysis" OR "cost utility analysis" OR "cost- benefit analysis" OR "cost-utility analysis" OR "cost and benefit" OR "cost and utility" OR "model revision" OR "usefulness" OR "performance" OR "utility" | Title, abstract, keywords | |
| Concept: Evaluation strategy | |  | |
| Strings | "in silico" OR "computer simulation" OR "simulation" OR "digital twin" OR "digital evaluation" OR "digital simulation" OR "computational simulation" OR "computational evaluation" OR "computational model" OR "pre-implementation" OR "pre-deployment" | Title, abstract, keywords | |
| Pubmed, Search date: 19/05/2023 | | | |
| Concept: CDS models, algorithms, and systems | |  | |
| Strings | "machine learning"[Text Word] OR "deep learning"[Text Word] OR "artificial intelligence"[Text Word] OR "reinforcement learning"[Text Word] OR "supervised learning"[Text Word] OR "unsupervised learning"[Text Word] OR "supervised machine learning"[Text Word] OR "unsupervised machine learning"[Text Word] OR "semi-supervised machine learning"[Text Word] OR "self-supervised machine learning"[Text Word] OR "semi-supervised learning"[Text Word] OR "self-supervised learning"[Text Word] OR "expert system"[Text Word] | Text Word | |
| MeSH | "Unsupervised Machine Learning"[Mesh] OR "Supervised Machine Learning"[Mesh] OR "Deep Learning"[Mesh] OR "Machine Learning"[Mesh] OR "Artificial Intelligence"[Mesh] |  | |
| Concept: Objective of the CDS model | |  | |
| Strings | "clinical decision support"[Text Word] OR "clinical decision making"[Text Word] OR "diagnos*"[Text Word] OR "prognos*"[Text Word] OR "screening"[Text Word] OR "triage"[Text Word] | Text Word | |
| MeSH | Decision Support Systems, Clinical[Mesh] OR Clinical Decision Rules[Mesh] OR Decision Support Techniques[Mesh] OR Prognosis[Mesh] OR Diagnosis[Mesh] OR Triage[Mesh] |  | |
| Concept: Evaluation objective | |  | |
| Strings | "credibility"[Text Word] OR "validation"[Text Word] OR "verification"[Text Word] OR "potential impact"[Text Word] OR "impact assessment"[Text Word] OR "decision analysis"[Text Word] OR "decision analytic measure*"[Text Word] OR "decision analytic model*"[Text Word] OR "net benefit"[Text Word] OR "decision curve*"[Text Word] OR "calibration"[Text Word] OR "model tuning"[Text Word] OR "credibility"[Text Word] OR "cost benefit analysis"[Text Word] OR "cost utility analysis"[Text Word] OR "cost-benefit analysis"[Text Word] OR "cost-utility analysis"[Text Word] OR "cost and benefit"[Text Word] OR "cost and utility"[Text Word] OR "model revision"[Text Word] OR "usefulness"[Text Word] OR "performance"[Text Word] OR "utility"[Text Word] | Text Word | |
| MeSH | Cost-Benefit Analysis[Mesh] OR Cost-effectiveness Analysis [Mesh] OR Software Validation[Mesh] OR Calibration[Mesh] |  | |
| Concept: Evaluation strategy | |  | |
| Strings | "in silico"[Text Word] OR "computer simulation*"[Text Word] OR "simulation*"[Text Word] OR "digital twin"[Text Word] OR "digital evaluation*"[Text Word] OR "digital simulation*"[Text Word] OR "computational simulation"[Text Word] OR "computational evaluation"[Text Word] OR "computational model*"[Text Word] OR "pre-implementation"[Text Word] OR "pre-deployment"[Text Word] | Text Word | |
| MeSH | Computer Simulation[Mesh] |  | |
| Other filters | |  | |
| Article types | clinicalstudy[Filter] OR clinicaltrial[Filter] OR clinicaltrialphasei[Filter] OR clinicaltrialphaseii[Filter] OR clinicaltrialphaseiii[Filter] OR clinicaltrialphaseiv[Filter] OR comparativestudy[Filter] OR controlledclinicaltrial[Filter] OR evaluationstudy[Filter] OR multicenterstudy[Filter] OR observationalstudy[Filter] OR pragmaticclinicaltrial[Filter] OR preprint[Filter] OR randomizedcontrolledtrial[Filter] OR validationstudy[Filter] |  | |
| Web of Science, Search date: 19/05/2023 | | | |
| Concept: CDS models, algorithms, and systems | |  | |
| Strings | “machine learning” OR “deep learning” OR “artificial intelligence” OR “reinforcement learning” OR “supervised learning” OR “unsupervised learning” OR “supervised machine learning” OR “unsupervised machine learning” OR “semi-supervised machine learning” OR “self-supervised machine learning” OR “semi-supervised learning” OR “self-supervised learning” OR “expert system” | Title, abstract, author keywords | |
| Concept: Objective of the CDS model | |  | |
| Strings | "clinical decision support" OR "clinical decision making" OR "diagnosis" OR "prognosis" OR "diagnoses" OR "prognoses" OR "diagnostic" OR "prognostic" OR "screening" OR "triage" | Title, abstract, author keywords | |
| Concept: Evaluation objective | |  | |
| Strings | "credibility" OR "validation" OR "verification" OR "potential impact" OR "impact assessment" OR "decision analysis" OR "decision analytic measure" OR "decision analytic model" OR "net benefit" OR "decision curve" OR "decision curve analysis" OR "calibration" OR "model tuning" OR "credibility" OR "cost benefit analysis" OR "cost utility analysis" OR "cost-benefit analysis" OR "cost-utility analysis" OR "cost and benefit" OR "cost and utility" OR "model revision" OR "usefulness" OR "performance" OR "utility" | Title, abstract, author keywords | |
| Concept: Evaluation strategy | |  | |
| Strings | "in silico" OR "computer simulation" OR "simulation" OR "digital twin" OR "digital evaluation" OR "digital simulation" OR "computational simulation" OR "computational evaluation" OR "computational model" OR "pre-implementation" OR "pre-deployment" | Title, abstract, author keywords | |
| Other filters | |  | |
| Article types | Review Article (Exclude – Document Types) and Editorial Material or Book Chapters or Database Review (Exclude – Document Types) |  | |
| IEEEXplore, Search date: 19/05/2023 | | | |
| Concept: CDS models, algorithms, and systems | |  | |
| Strings | “machine learning” OR “deep learning” OR “artificial intelligence” OR “reinforcement learning” OR “supervised learning” OR “unsupervised learning” OR “supervised machine learning” OR “unsupervised machine learning” OR “semi-supervised machine learning” OR “self-supervised machine learning” OR “semi-supervised learning” OR “self-supervised learning” OR “expert system” | Abstract, keywords | |
| Concept: Objective of the CDS model | |  | |
| Strings | "clinical decision support" OR "clinical decision making" OR "diagnosis" OR "prognosis" OR "diagnoses" OR "prognoses" OR "diagnostic" OR "prognostic" OR "screening" OR "triage" | Abstract, keywords | |
| Concept: Evaluation objective | |  | |
| Strings | "credibility" OR "validation" OR "verification" OR "potential impact" OR "impact assessment" OR "decision analysis" OR "decision analytic measure" OR "decision analytic model" OR "net benefit" OR "decision curve" OR "decision curve analysis" OR "calibration" OR "model tuning" OR "credibility" OR "cost benefit analysis" OR "cost utility analysis" OR "cost-benefit analysis" OR "cost-utility analysis" OR "cost and benefit" OR "cost and utility" OR "model revision" OR "usefulness" OR "performance" OR "utility" | Abstract, keywords | |
| Concept: Evaluation strategy | |  | |
| Strings | "in silico" OR "computer simulation" OR "simulation" OR "digital twin" OR "digital evaluation" OR "digital simulation" OR "computational simulation" OR "computational evaluation" OR "computational model" OR "pre-implementation" OR "pre-deployment" | Abstract, keywords | |
| Other filters | |  | |
| Article types | Conference proceedings title page and front cover (Exclude – Document Types) |  | |
| Article types | Books and magazines (Exclude – Document Types) |  | |
| Article types | Journals, Early Access, and Conference articles (Incude – Document Types) |  | |
| Cochrane, Search date: 19/05/2023 | | | |
| Concept: CDS models, algorithms, and systems | |  | |
| Strings | (“machine learning” OR “deep learning” OR “artificial intelligence” OR “reinforcement learning” OR “supervised learning” OR “unsupervised learning” OR “supervised machine learning” OR “unsupervised machine learning” OR “semi-supervised machine learning” OR “self-supervised machine learning” OR “semi-supervised learning” OR “self-supervised learning” OR “expert system”):ti,ab,kw (Word variations have been searched)] | Title, abstract, keywords | |
| MeSH | "Unsupervised Machine Learning"[Mesh] OR "Supervised Machine Learning"[Mesh] OR "Deep Learning"[Mesh] OR "Machine Learning"[Mesh] OR "Artificial Intelligence"[Mesh] | Explode all trees | |
| Concept: Objective of the CDS model | |  | |
| Strings | ("clinical decision support" OR "clinical decision?making" OR "diagnos*" OR "prognos*" OR "screening" OR "triage"):ti,ab,kw (Word variations have been searched) | Title, abstract, keywords | |
| MeSH | Decision Support Systems, Clinical[Mesh] OR Clinical Decision Rules[Mesh] OR Decision Support Techniques[Mesh] OR Prognosis[Mesh] OR Diagnosis[Mesh] OR Triage[Mesh]) | Explode all trees | |
| Concept: Evaluation objective | |  | |
| Strings | ("credibility" OR "validation" OR "verification" OR "potential impact" OR "impact assessment" OR "decision analysis" OR "decision analytic measure*" OR "decision analytic model*" OR "net benefit" OR "decision curve*" OR "decision curve analysis" OR "calibration" OR "model tuning" OR "credibility" OR "cost?benefit analysis" OR "cost?utility analysis" OR "cost and benefit" OR "cost and utility" OR "model revision" OR "usefulness" OR "performance" OR "utility"):ti,ab,kw | Title, abstract, keywords | |
| MeSH | Economics, Pharmaceutical [Mesh] OR Cost-benefit Analysis[MeSH] OR Cost-effectiveness Analysis [Mesh] OR Software Validation[Mesh] OR Calibration[Mesh] | Explode all trees | |
| Concept: Evaluation strategy | |  | |
| Strings | ("in silico" OR "computer simulation*" OR "simulation*" OR "digital twin" OR "digital evaluation*" OR "digital simulation*" OR "computational simulation" OR "computational evaluation" OR "computational model*" OR "pre-implementation" OR "pre-deployment"):ti,ab,kw (Word variations have been searched) | Title, abstract, keywords | |
| MeSH | Computer Simulation[Mesh] | Explode all trees | |
| Other filters | |  | |
| Article types | English articles |  | |
| Arxiv, Search date: 19/05/2023 | | | |
| Concept: CDS models, algorithms, and systems | |  | |
| Strings | "machine learning" OR "deep learning" OR "artificial intelligence" OR "reinforcement learning" OR "supervised learning" OR "unsupervised learning" OR "supervised machine learning" OR "unsupervised machine learning" OR "semi-supervised machine learning" OR "self-supervised machine learning" OR "semi-supervised learning" OR "self-supervised learning" OR "expert system" | Title, abstract | |
| ACM | I.2 |  | |
| MSC | 68T |  | |
| Concept: Objective of the CDS model | |  | |
| Strings | "clinical decision support" OR "clinical decision?making" OR "diagnos*" OR "prognos*" OR "screening" OR "triage" | Title, abstract | |
| ACM | J.3 |  | |
| MSC | 62P10 |  | |
| Concept: Evaluation objective | |  | |
| Strings | "credibility" OR "validation" OR "verification" OR "potential impact" OR "impact assessment" OR "decision analysis" OR "decision analytic measure*" OR "decision analytic model*" OR "net benefit" OR "decision curve*" OR "decision curve analysis" OR "calibration" OR "model tuning" OR "credibility" OR "cost?benefit analysis" OR "cost?utility analysis" OR "cost and benefit" OR "cost and utility" OR "model revision" OR "usefulness" OR "performance" OR "utility" | Title, abstract | |
| ACM | C.4 OR C.5 |  | |
| MSC | 68M20 OR 68M15 |  | |
| Concept: Evaluation strategy | |  | |
| Strings | "in silico" OR "computer simulation*" OR "simulation*" OR "digital twin" OR "digital evaluation*" OR "digital simulation*" OR "computational simulation" OR "computational evaluation" OR "computational model*" OR "pre-implementation" OR "pre-deployment" | Title, abstract | |
| ACM | I.6.4 OR I.6.4 |  | |
| MSC | 68U20 |  | |
| Other filters | |  | |
| Max results | 10_000, highest possible search results |  | |

*Notes: MeSH: Medical Subject Headings; Emtree: Embase subject headings; ACM: Association for Computing Machinery Computing Classification System; MSC: Mathematics Subject Classification*

## Sample encoding sheet


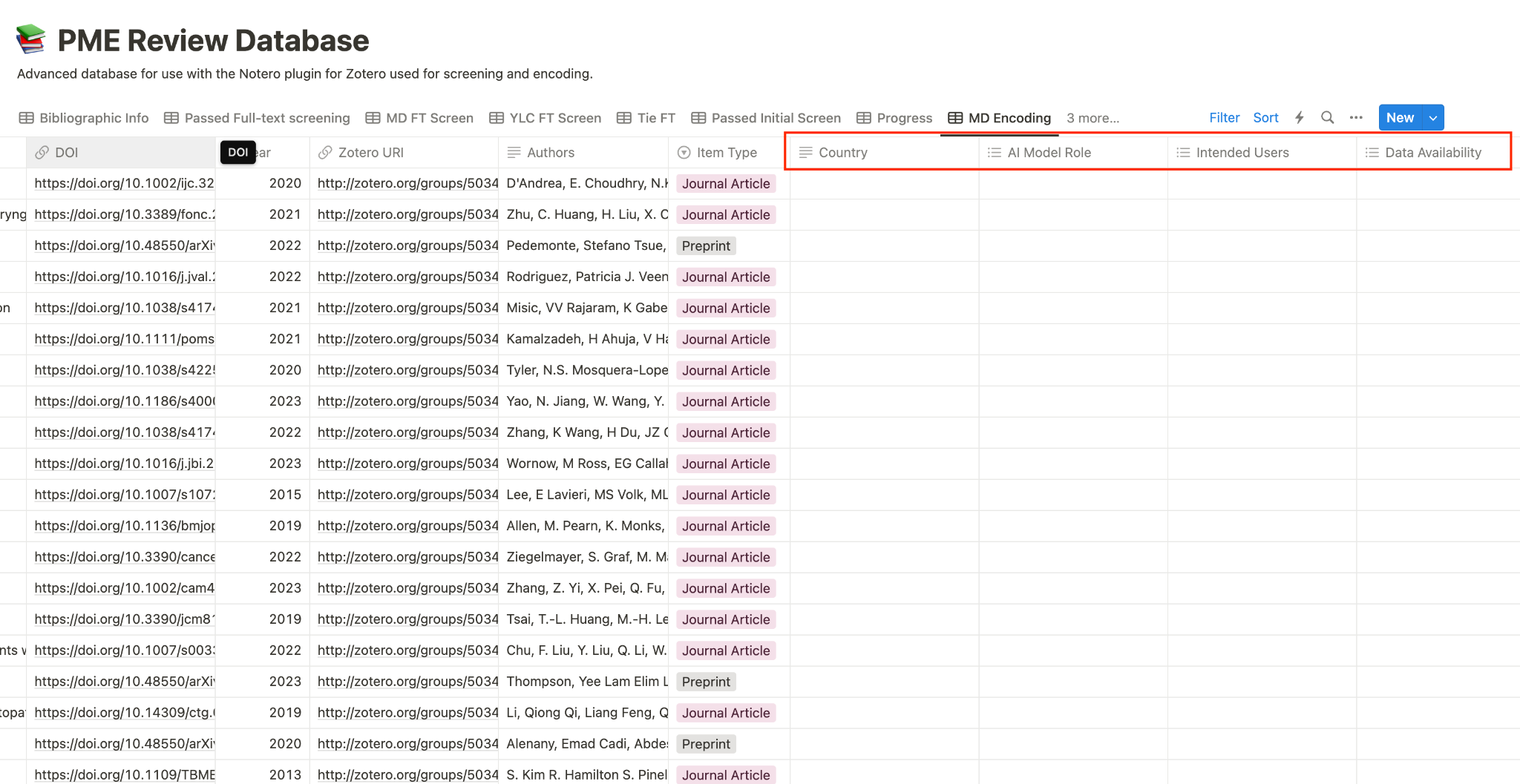


**Figure S1.** Screenshot of sample encoding sheet for 1 reviewer. Notion.so was used in the screening and encoding stages of the review. Emphasis was added for sample encoding items.
