# Supplementary figures and images for: In Silico Evaluation of Algorithm-Based Clinical Decision Support Systems Based on Care Pathway Simulation Models: Scoping Review
Source: JMIR AI. 2026 Mar 24;5:e72472. doi: 10.2196/72472 (PMC13012236; doi:10.2196/72472)

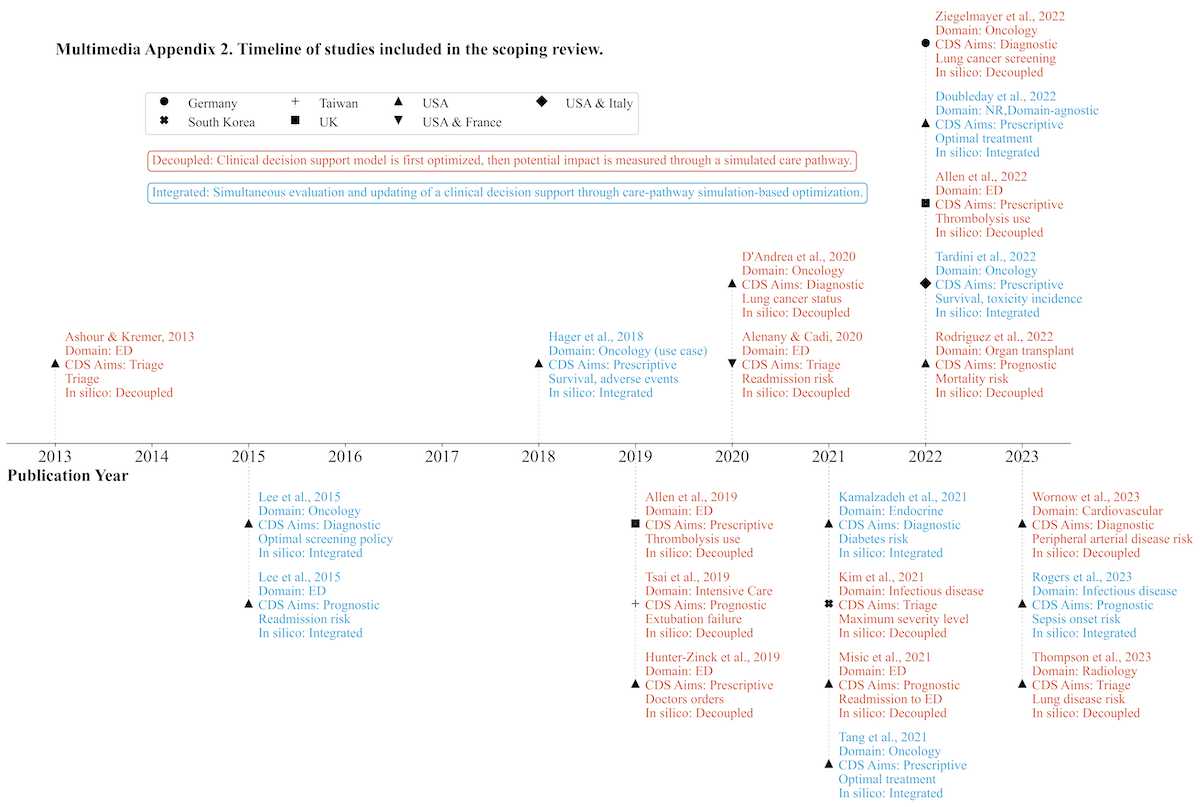

Supplement: Multimedia Appendix 2 [file ai-v5-e72472-s002.png]
